# Bioactivities by a crude extract from the Greenlandic *Pseudomonas* sp. In5 involves the nonribosomal peptides, nunamycin and nunapeptin

**DOI:** 10.7717/peerj.1476

**Published:** 2015-12-03

**Authors:** Charlotte F. Michelsen, Helle Jensen, Vincent J. Venditto, Rosanna C. Hennessy, Peter Stougaard

**Affiliations:** 1 Department of Systems Biology, Technical University of Denmark, Kgs. Lyngby, Denmark; 2 Department of Microbiology and Immunology, University of California, San Francisco, CA, United States; 3 Departments of Bioengineering and Therapeutic Sciences, School of Pharmacy, University of California, San Francisco, CA, United States; 4 Department of Plant and Environmental Sciences, University of Copenhagen, Frederiksberg C, Denmark

**Keywords:** Crude extract, Nonribosomal peptides, Antimicrobial, Anticancer, Pseudomonas

## Abstract

**Background.** Bioactive microbial metabolites provide a successful source of novel compounds with pharmaceutical potentials. The bacterium *Pseudomonas* sp. In5 is a biocontrol strain isolated from a plant disease suppressive soil in Greenland, which produces two antimicrobial nonribosomal peptides (NRPs), nunapeptin and nunamycin.

**Methods.** In this study, we used *in vitro* antimicrobial and anticancer bioassays to evaluate the potential bioactivities of both a crude extract derived from *Pseudomonas* sp. In5 and NRPs purified from the crude extract.

**Results.** We verified that the crude extract derived from *Pseudomonas* sp. In5 showed suppressive activity against the basidiomycete *Rhizoctonia solani* by inducing a mitochondrial stress-response. Furthermore, we confirmed suppressive activity against the oomycete *Pythium aphanidermatum* by the *Pseudomonas* sp. In5 crude extract, and that the purified nunamycin and nunapeptin displayed distinct antimicrobial activities. In addition to the antimicrobial activity, we found that treatment of the cancer cell lines, Jurkat T-cells, Granta cells, and melanoma cells, with the *Pseudomonas* sp. In5 crude extract increased staining with the apoptotic marker Annexin V while no staining of healthy normal cells, i.e., naïve or activated CD4 T-cells, was observed. Treatment with either of the NRPs alone did not increase Annexin V staining of the Jurkat T-cells, despite individually showing robust antimicrobial activity, whereas an anticancer activity was detected when nunamycin and nunapeptin were used in combination.

**Discussion.** Our results suggest that the bioactivity of a crude extract derived from *Pseudomonas* sp. In5 involves the presence of both nunamycin and nunapeptin and highlight the possibility of synergy between multiple microbial metabolites.

## Introduction

Microorganisms have been used as a privileged source of bioactive metabolites with pharmaceutical properties, such as antifungal, antibacterial, antiviral, anticancer or immunosuppressive activities, since the successful discovery of penicillin more than 80 years ago (Cameotra & Makkar, 2004; Demain & Sanchez, 2009). Consequently, many pharmaceutical drugs used today are either naturally derived bioactive metabolites or inspired thereof (Demain & Sanchez, 2009; Felnagle et al., 2008).

Nonribosomal peptides (NRPs) are a class of secondary metabolites, which are produced mainly by microorganisms with large genomes. NRPs show an enormous structural diversity due to incorporation of nonstandard amino acids and have a broad range of biological activities, including pharmaceutical properties (Donadio, Monciardini & Sosio, 2007; Felnagle et al., 2008; Jokela et al., 2012; Pirri et al., 2009; Raaijmakers, De Bruijn & De Kock, 2006; Shishido et al., 2015). An example of an NRP with great pharmaceutical potential is romidepsin (also known as FK228 or FR901228). Romidepsin is produced by *Chromobacterium violaceum* and displays potent antimicrobial and anticancer activities (Cheng, Yang & Matter, 2007; Skov et al., 2005; Valdez et al., 2015). In 2009, romidepsin was approved by the US FDA for treatment of cutaneous T-cell lymphoma (VanderMolen et al., 2011) and was introduced to the market in 2010 (Newman & Cragg, 2012). There is a continuous demand for research to identify and develop new classes of chemotherapeutic agents, due to evolvement of drug resistance and common side effects such as lack of specificity towards the cancer cells (Gottesman, 2002; Lake & Robinson, 2005), and natural bioactive metabolites, such as NRPs, could provide an important source for new drug candidates. However, not all NRPs derived from microorganisms show anticancer activity. A recent study by Caboche et al. (2010) predicted that approximately 4% of the NRPs listed in the Norine Database have anticancer potential based on the individual structure of the compounds.

The traditional workflow to discover bioactive metabolites as potential drug candidates is the use of biobased isolation strategies, in which chemical profiles of crude extracts are combined with activity data from *in vitro* bioassays, followed by purification and investigation of individual compounds (Singh & Pelaez, 2008). However, it is important to note that most crude extracts are comprised of multiple bioactive metabolites. Thus, purification and investigation of individual metabolites will not detect bioactivities, which involve a synergy of multiple compounds.

**Figure 1:**
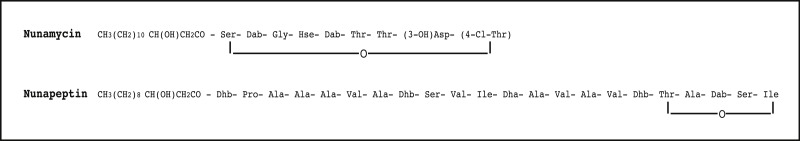
The proposed structure of the two nonribosomal peptides, nunamycin and nunapeptin, from *Pseudomonas* sp. In5. The proposed structure of nunamycin (*m*/*z* 1,138) and nunapeptin (*m*/*z* 2,023–2,075) from *Pseudomonas* sp. In5 (Michelsen et al., 2015). The abbreviations are (3-OH)Asp, 3-hydroxyaspartic acid; (4-Cl)Thr, 4-Chlorothreonine; Dab, 2,4-Diaminobutyric Acid; Dhb, Dehydrobutyrine; Dha, Dehydroalanine; Orn, ornithine and Hse, homoserine.

We have previously isolated the biocontrol bacterium *Pseudomonas* sp. In5 from a Greenlandic potato soil, which possesses strong antimicrobial activity towards various filamentous fungi and oomycetes in both *in vitro* dual-culture inhibition assays and soil microcosms (Michelsen & Stougaard, 2011; Michelsen & Stougaard, 2012; Michelsen et al., 2015). Two NRPs, i.e., nunamycin and nunapeptin, have been isolated from *Pseudomonas* sp. In5 using a biobased isolation strategy and showed distinct antimicrobial activities (Michelsen et al., 2015). Further investigations into the structures of the two NRPs revealed that nunamycin is a monochlorinated nine amino acid cyclic lipopeptide, whereas nunapeptin is a 22 amino acid cyclic lipopeptide (shown in Fig. 1) (Michelsen et al., 2015). In this study, we are expanding our knowledge of the potential bioactivities of *Pseudomonas* sp. In5 using a crude extract, which contains the NRPs nunamycin and nunapeptin. We found that the crude extract from *Pseudomonas* sp. In5, possessed strong antimicrobial activity against the basidiomycete *R. solani* Ag3 and the oomycete *P. aphanidermatum*, confirming the observations in our previous study (Michelsen et al., 2015). In addition, the *Pseudomonas* sp. In5 derived crude extract showed potent anticancer activity in that it was able to increase staining of T-cell leukemia, mantle cell lymphoma, and melanoma cell lines with the apoptotic marker Annexin V, while not affecting normal healthy cells, i.e., naïve or activated CD4 T-cells. When examining the bioactivities of nunamycin and nunapeptin, two NRPs purified from the crude extract, we found that anticancer activity was only observed when the two NRPs were used in combination, despite showing individual antimicrobial activities. Together these results highlight that the bioactivities of a microbial crude extract may involve either single metabolites or a synergy between multiple metabolites.

## Materials and Methods

### Bacterial and fungal strains

The *Pseudomonas* sp. strains, In5 (wild type) and Gr1 (nunamycin knock-out mutant described in Michelsen et al. (2015)), were routinely grown in liquid or on solid (1.5% agar) Luria-Bertani medium at 20 °C. For strain Gr1, the medium was supplemented with kanamycin (50 µg/ml). The basidiomycete *R. solani* Ag3 (provided by LKF Vandel, Denmark) and the oomycete *P. aphanidermatum* (provided by the Section for Microbial Ecology and Biotechnology, Department of Plant and Environmental Sciences, Faculty of Sciences, University of Copenhagen) were used as target strains in the *in vitro* antimicrobial assay. *R. solani* and *P. aphanidermatum* were maintained on standard potato dextrose agar (PDA) medium at 20 °C.

### Cancer cell lines

The cancer cell lines, Jurkat T-cells (T-cell leukemia), Granta cells (mantle cell lymphoma), and the melanoma cell line FM-86 were kindly provided by Dr. Søren Skov, Section of Experimental Animal Models, Faculty of Health and Medical Sciences, University of Copenhagen, Denmark. The cells were grown in RPMI-1640 medium (Sigma-Aldrich, St. Louis, MO, USA) supplemented with 10% FBS (Sigma-Aldrich, St. Louis, MO, USA), 2 mmol/L glutamine (Sigma-Aldrich, St. Louis, MO, USA), penicillin (100 IU)/streptomycin (100 µg/ml) (Sigma-Aldrich, St. Louis, MO, USA). All cells were incubated at 37 °C and 5% CO_2_ and passaged every second and third day.

### Preparation of crude extracts

Plates with 1/5 PDA medium were inoculated with *Pseudomonas* sp. In5 or the nunamycin mutant, Gr1. After incubation for 48 h at room temperature, the agar was sliced into small pieces and extracted with acidified deionized water (pH 2) for 1 h at 20 °C and 225 rpm in a 2.8-liter Erlenmeyer flask followed by addition of n-butanol and incubation for 18 h at 20 °C as previously described (Michelsen et al., 2015). The n-butanol extract was separated from agar by cheesecloth filtration and subsequently from cell debris by centrifugation (6,440 × g, 10 min). n-butanol was removed with a rotary evaporator (Buechi R-200).

The presence of the two NRPs, nunamycin (*m*/*z* 1,138) and nunapeptin (i.e., the nunapeptin family of *m*/*z* 2,023–2,075), in the extracts was evaluated by matrix-assisted laser desorption/ionization—time-of-flight mass spectrometry (MALDI-TOF MS) analysis as described by Liu et al. (2010). n-butanol was removed with a rotary evaporator (Maxi Dry Lyo; Holm & Halby, Brøndby, Denmark) and the dry extracts were stored at −20 °C until further use.

### Purification of nunamycin and nunapeptin

Nunamycin and nunapeptin were purified from the *Pseudomonas* sp. In5 derived extract by size-exclusion chromatography using a Sephadex™ LH-20 column followed by high performance liquid chromatography as previously described (Michelsen et al., 2015). In brief, the *Pseudomonas* sp. In5 derived extract was dissolved in 1 mL methanol and eluted through a 0.7 × 30 cm column containing Sephadex LH20 resin equilibrated in methanol. The first 7.5 mL of methanol was discarded as waste and the next 10 mL were collected and concentrated. The resulting brown residue was taken up in 200 µL of methanol and purified by reverse phase HPLC on a Vydac C18 column with 0.1% trifluoroacetic acid in water (solvent A) and 0.1% trifluoroacetic acid in acetonitrile (solvent B). The gradient started with a 5 min hold in 10% B followed by a 45 min linear ramp to 90% B. Nunamycin eluted at 27.5 min and nunapeptin eluted as a group of peaks between 34 and 37 min. The presence of each compound was confirmed by MALDI-TOF MS. Respective fractions from HPLC were combined and lyophilized to yield 1 mg of each compound. The purified compounds were stored at −20 °C until further use.

### *In vitro* antimicrobial assay

For analyzing the antimicrobial activity of extracts or purified NRPs, 10 µg solid/ml of the *Pseudomonas* sp. In5 or Gr1 derived extracts or 10 µg of the purified nunamycin and nunapeptin compounds was added to a sterile filter disc in the center of a 1/5 strength PDA plate. Four 6 mm plugs of target fungal or oomycete mycelia were placed 2 cm from the center of the plate. 23% EtOH in Milli-Q H_2_O was used as negative control. Plates were incubated at 20 °C and antimicrobial activity was determined as percent inhibition of radial growth (PIRG) as described by Whipps (1987) after 3 days of incubation. Tukey’s HSD test was used in conjunction with ANOVA in order to evaluate which means were significantly different from one another. P-values are considered significant at *p* < 0.05. The tests were carried out with R version 3.0.3 (R Development Core Team, 2009). When applicable, the data are presented as mean ± SEM.

### Microscopy of *R. solani* Ag3 fungal hyphae

A thin layer of 1/10 strength PDA was spread on top of a 76 × 26 mm sterile object glass (Menzel-Gläser, Braunschweig, Germany) in sterile Petri dishes. Two µg solid/ml of the *Pseudomonas* sp. In5 derived extract was added 2 cm away from a fungal mycelia plug placed in the center of the slide and incubated at 28 °C for 48 h. Slides containing fungal hyphae treated with four µl of 23% EtOH in Milli-Q H_2_O served as control treatments. The experiment was carried out in triplicates. Following incubation, the slides were cut out of the Petri dishes and placed in a Zeiss Axioplan 2 Imaging fluorescence microscope (Brock & Michelsen A/S, Birkerød, Denmark). Preparations were visualized by 40× magnification and recorded with the AxioCam MRc program. In order to perform vital staining of mitochondria in *R. solani* Ag3 fungal hyphae with the fluorescent dye, DiOC_7_(3) (3, 3′-Diheptyloxacarbocyanine iodide) (Duckett & Read, 1991), 1 µg/ml of DiOC_7_(3) was added to the samples and incubated for 5 min at 30 °C before visualization by fluorescence microscopy (Zeiss Axioplan 2 Imaging fluorescence microscope (Brock & Michelsen A/S, Birkerød, Denmark)).

### Isolation and stimulation of CD4 T-cells

CD4 T-cells were isolated from peripheral blood mononuclear cells obtained from healthy blood donors (The State Hospital, Copenhagen, Denmark) using CD4 microbeads (Miltenyi Biotec, Bergisch Gladbach, Germany). The CD4 T-cells were left untouched (naïve) or activated with one Dynabead CD3/CD28 T-cell expander (Invitrogen, Carlsbad, CA, USA) per cell for three days in RPMI-1640 medium, supplemented with 5% human serum (Lonza, Basel Switzerland), 2 mmol/L glutamine, 2 mmol/L penicillin/streptomycin.

### Detection of Annexin V and PI staining by flow cytometry

For the bacterial extracts: the cancer cells (i.e., Jurkat T-cells, Granta cells, and the melanoma cells) as well as naïve CD4 T-cells, or activated CD4 T-cells were incubated with 23% EtOH in Milli-Q H_2_O (control) at a final dilution of 1:200, the anticancer agent, FR901228 (20 ng/ml, obtained from the National Cancer Institute, Bethesda, MD), *Pseudomonas* sp. In5 (1.5 or 2.5 µg solid/ml) or *Pseudomonas* sp. Gr1 (2.5 µg solid/ml) derived crude extracts for 18 h at 37 °C and 5% CO_2_. For the purified nunamycin and nunapeptin compounds: Jurkat T-cells were incubated with DMSO (control) or 10 µg/ml compounds either alone or in combination for 24 h at 37 °C and 5% CO_2_. Cells were stained with FITC-labeled Annexin V (BD Biosciences, Franklin Lakes, NJ, USA) according to the manufactures protocol. PI staining of cells was done by adding 1 µg/ml propidium iodide (PI, Sigma-Aldrich, St. Louis, MO, USA) to the samples followed by incubation at room temperature for 1 min. Data acquisition was done on a BD LSR II or Accuri C6. Data was analyzed using FlowJo software. All group comparisons were performed using a Students *t*-test. P-values are considered significant at *p* < 0.05. When applicable, the data are presented as mean ± SEM.

## Results

### A crude extract derived from Pseudomonas sp. In5 possesses strong antimicrobial activity by inducing a mitochondrial stress-response

**Figure 2:**
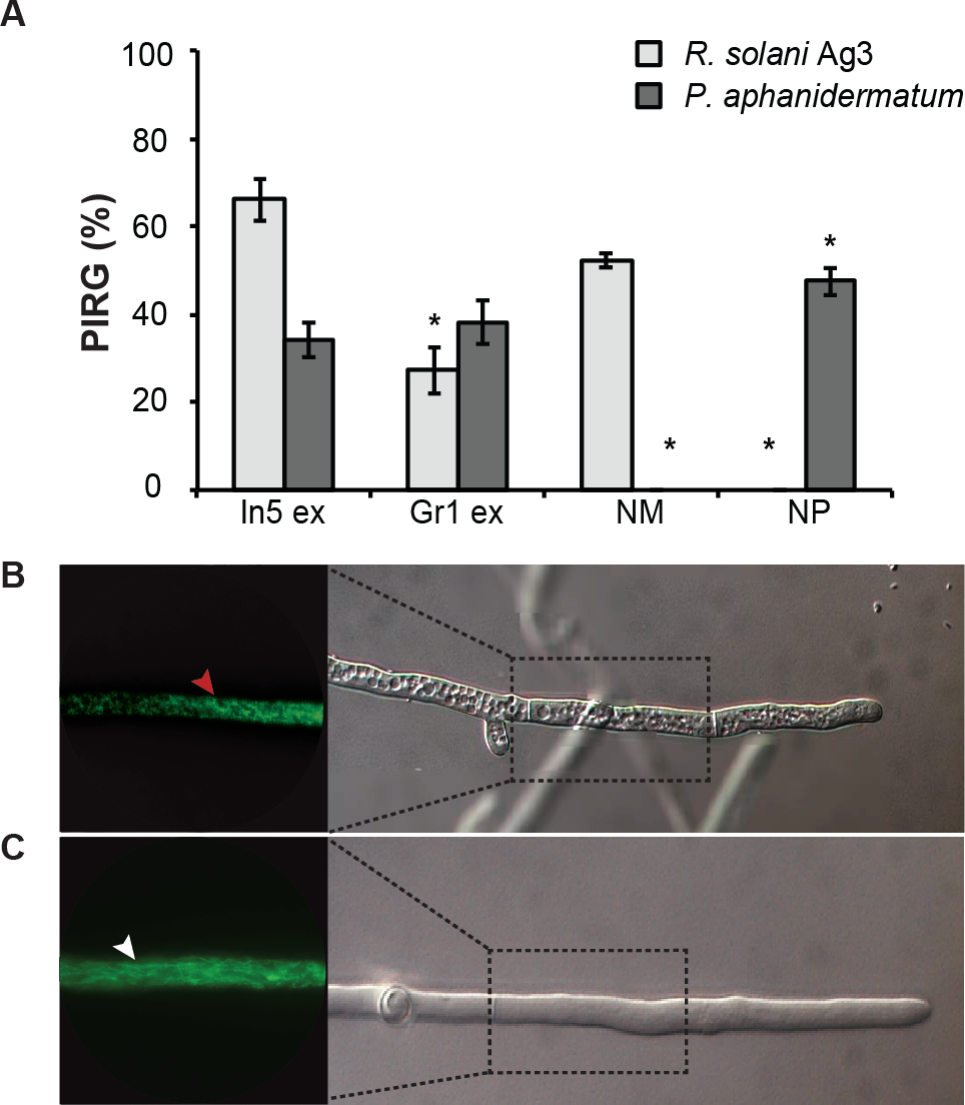
Antimicrobial activities of *Pseudomonas* crude extracts and purified nonribosomal peptides. (A) The antimicrobial effect of the crude extracts derived from wild type *Pseudomonas* sp. In5 (In5 ex) or the nunamycin knock-out mutant, Gr1 (Gr1 ex) as well as the purified NRPs, nunamycin (NM) or nunapeptin (NP) was examined against the basidiomycete, *R. solani* AG3 (light grey bars), or oomycete, *P. aphanidermatum* (dark grey bars). Antimicrobial activity was scored as percent inhibition of radial growth (PIRG) (mean ± SEM, *n* = 3). Asterisk indicates statistically significance in relation to the In5 extract (*p* < 0.05, Tukey’s HSD test). The antifungal effect by the NRP extract derived from wild type *Pseudomonas* sp. In5 (B) compared to the control treatment, 23% EtOH in Milli-Q H_2_O (C) toward *R. solani* Ag3 hyphae was examined near the zone of inhibition by microscopy (*n* = 3). Staining with DiOC_7_(3) revealed small, randomly organized mitochondria in the NRP extract treated hyphae (red arrowhead) compared to longitudinal shaped mitochondria in the control treatment (white arrowhead).

**Figure 3:**
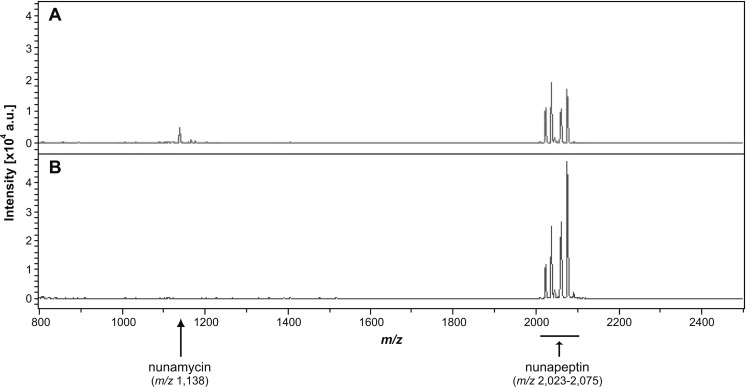
Identification of nonribosomal peptides in *Pseudomonas* crude extracts by MALDI-TOF MS. Identification of nunamycin and/or nunapeptin from *Pseudomonas* sp. In5 or Gr1 derived crude extracts. The average signals from *m*/*z* 800 to 2,500 of the spectra obtained from MALDI-TOF Mass Spectrometry of the extract from wild type *Pseudomonas* sp. In5 (A) and the nunamycin knock-out mutant, Gr1 (B). Arrows indicate the signals of nunamycin (*m*/*z* 1,138) and nunapeptin (*m*/*z* 2,023–2,075).

We have previously shown that the Greenlandic biocontrol bacterium *Pseudomonas* sp. In5 possesses strong antimicrobial activity against various filamentous fungi and oomycetes by using *in vitro* co-culture experiments with either bacterium, extract or the purified NRPs nunamycin and nunapeptin (Michelsen & Stougaard, 2011; Michelsen & Stougaard, 2012; Michelsen et al., 2015). To expand our knowledge about the pharmaceutical potential of the bioactive metabolites derived from *Pseudomonas* sp. In5 we prepared a crude extract by organic solvent extraction and tested the extract for antimicrobial activity using an *in vitro* agar diffusion assay. The crude extract exhibited strong antimicrobial activities with 66% and 34% mean inhibition of mycelial radial growth of *R. solani* Ag3 and *P. aphanidermatum*, respectively (Fig. 2A), which was consistent to the activities previously observed with *Pseudomonas* sp. In5 (Michelsen & Stougaard, 2011; Michelsen & Stougaard, 2012; Michelsen et al., 2015). A more detailed microscopic analysis of the *in vitro* effect of the crude extract against *R. solani* hyphae was employed by vital staining of mitochondria with the fluorescent dye DiOC_7_(3) (Duckett & Read, 1991). Notably, treatment with the *Pseudomonas* sp. In5 derived crude extract caused changes in growth and cell morphology of the *R. solani* hyphae, including intense vacuolization in the hyphae tips and increased branching and swelling of the hyphae (Fig. 2B). In contrast, the control treatment did not show any abnormal morphology of the *R. solani* hyphae (Fig. 2C). Furthermore, intracellular activities of mitochondria in the hyphae treated with the crude extract were reduced compared to the control treatment and the mitochondria became randomly organized with an altered morphology from longitudinal shaped (i.e., control treatment) to small and circular (i.e., In5 extract treatment) (Figs. 2C and 2B, respectively). The crude extract was analyzed by MALDI-TOF MS and confirmed the presence of the two antimicrobial NRPs, nunamycin (*m*/*z* 1,138) and nunapeptin (i.e., the nunapeptin family of *m*/*z* 2,023–2,075) (Fig. 3A).

We additionally prepared a crude extract from the mutant strain *Pseudomonas* sp. Gr1, which is deficient in producing the NRP, nunamycin (Michelsen et al., 2015). As confirmed by MALDI-TOF MS analysis, nunapeptin, but not nunamycin, was detected in the *Pseudomonas* sp. Gr1 derived crude extract (Fig. 3B). When tested for antimicrobial activity this crude extract showed decreased antimicrobial activity against *R. solani* with a mean value of 27% inhibition of mycelial radial growth (Fig. 2A),  whereas the activity against *P. aphanidermatum* was comparable to that observed with the *Pseudomonas* sp. In5 derived crude extract (i.e., mean value of 38% and 34% inhibition, respectively) (Fig. 2A). These results are consistent with our previous results obtained from co-culture experiments with the *Pseudomonas* sp. Gr1 strain (Michelsen et al., 2015) and suggest that the strong antimicrobial effect against *R. solani* by the *Pseudomonas* sp. In5 derived crude extract involves the activity of nunamycin as well as nunapeptin and/or other metabolites present in the extract, whereas the antimicrobial effect against *P. aphanidermatum* is not dependent on nunamycin.

### The antimicrobial crude extract derived from Pseudomonas sp. In5 increases Annexin V staining of cancer cells, but not of naïve or activated T-cells

**Figure 4:**
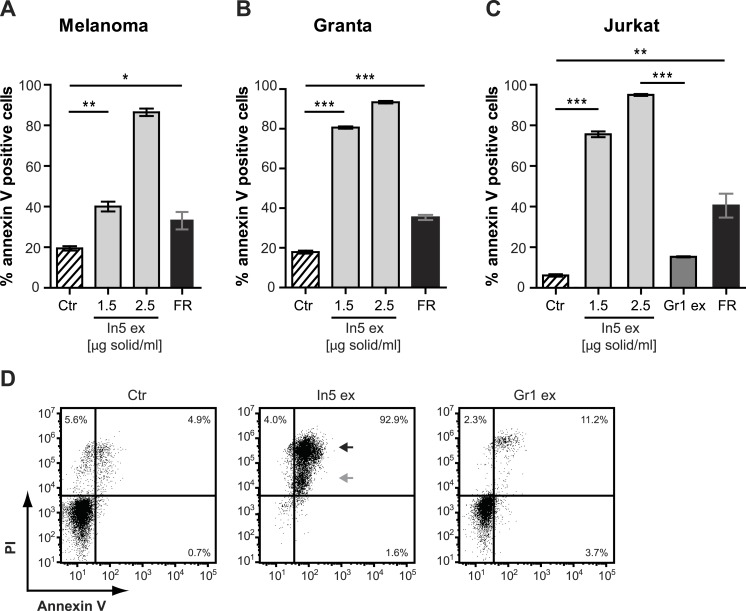
Annexin V staining of cancer cell lines after treatment with *Pseudomonas* crude extracts. Annexin V staining of (A) melanoma, (B) Granta, and (C) Jurkat T-cell cancer cell lines was examined by flow cytometry after treatment with 23% EtOH in Milli-Q H_2_O at a final dilution of 1:200 (control, Ctr), the well-known anticancer agent, FR901228 (FR, 20 ng/ml), or crude extracts derived from *Pseudomonas* sp. In5 (In5 ex, 1.5 or 2.5 µg solid/ml) or Gr1 (Gr1 ex, 2.5 µg solid/ml) (mean ± SEM; *n* = 4). ^∗^*p* < 0.05; ^∗∗^*p* < 0.01; ^∗∗∗^*p* < 0.001 (students *t*-test). (D) Annexin V and PI staining of Jurkat T-cells was examined by flow cytometry after treatment with 23% EtOH in Milli-Q H_2_O at a final dilution of 1:200 (control, Ctr) or crude extracts derived from *Pseudomonas* sp. In5 (In5 ex, 2.5 µg solid/ml) or Gr1 (Gr1 ex, 2.5 µg solid/ml). The grey arrowhead shows late apoptotic cells (Annexin V^+^, PI^dim^), whereas the black arrowhead shows late apoptotic/necrotic cells (Annexin V^+^, PI^bright^). The *Y*-axis shows PI staining (in fluorescence units) and the *X*-axis shows Annexin V staining (in fluorescence units), both on a log scale. Data is representative of four independent experiments.

**Figure 5:**
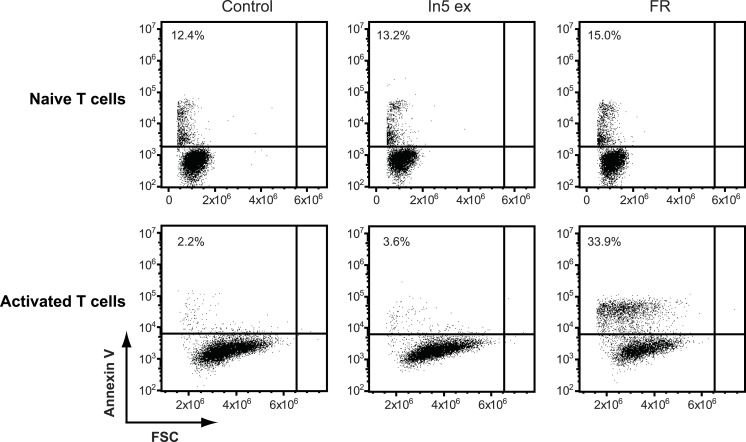
Annexin V staining of naïve or CD3/CD28 stimulated CD4 T cells after treatment with *Pseudomonas* crude extract. Annexin V staining of naïve or CD3/CD28 stimulated CD4 T cells was examined by flow cytometry after treatment with 23% EtOH in Milli-Q H_2_O at a final dilution of 1:200 (control), FR901228 (FR, 20 ng/ml), or 1.5 µg solid/ml of *Pseudomonas* sp. In5 derived crude extract (In5 ex) (*n* = 4). The *Y*-axis shows Annexin V staining (in fluorescence units) in log scale and the *X*-axis shows cell size as Forward Scatter (FSC) in linear scale.

The antimicrobial crude extract derived from *Pseudomonas* sp. In5 was further analyzed for anticancer activity by testing the ability to induce Annexin V staining in three different cancer cell lines, i.e., the Jurkat T-cell line (T-cell leukemia), the Granta cell line (mantle cell lymphoma), and a melanoma cell line. In cells undergoing apoptosis, loss of cell membrane asymmetry is one of the earliest morphological features, which causes the membrane phospholipid phosphatidylserine (PS) to translocate from the inner to the outer leaflet of the cell membrane and thereby exposes it to the extracellular environment. Annexin V is a phospholipid-binding protein that binds PS with high affinity and can therefore be used to detect cells undergoing apoptosis (Vermes et al., 1995). Interestingly, a significant increase of Annexin V staining of all three cancer cell lines tested was observed with the In5 derived crude extract compared to the control treatment (Figs. 4A–4C). A mean value of 40.1% (melanoma cells), 80.7% (Granta cells), or 75.6% (Jurkat T-cells) Annexin V positive cells was observed at a concentration of 1.5 µg solid/ml (Figs. 4A–4C, respectively), which was further increased at a concentration of 2.5 µg solid/ml (Figs. 4A–4C). Numerous chemotherapeutic drugs target proliferating cells and hence can affect the viability of cancer cells as well as normal healthy cells (Lake & Robinson, 2005). We therefore extended our analysis of the anticancer potential and examined the effect of the *Pseudomonas* sp. In5 derived crude extract on non-proliferating naïve and proliferating CD3/CD28 stimulated CD4 T-cells. Strikingly, the crude extract did not induce Annexin V staining of neither naïve nor activated CD4 T-cells (Fig. 5). In contrast, treatment with the known anticancer agent FR901228 (VanderMolen et al., 2011) induced Annexin V staining of all the cancer cells tested (Figs. 4A–4C) as well as activated CD4 T-cells, but not in naïve CD4 T-cells (Fig. 5). Together these results suggest that the *Pseudomonas* sp. In5 derived crude extract contain one or more metabolites with anticancer potential.

Since a robust induction of Annexin V staining was observed for all three cancer cell lines tested, the Jurkat T-cell line was selected for further investigations of anticancer activity by the *Pseudomonas* sp. In5 crude extract. We included PI staining in our flow cytometry analysis of Jurkat T-cells treated with 2.5 µg solid/ml of the *Pseudomonas* sp. In5 crude extract and found that almost all cells were double positive for Annexin V and PI staining (i.e., Annexin V^+^PI^+^) (Fig. 4D). Necrotic cells or late apoptotic cells undergoing secondary necrosis (Silva, 2010) are quickly stained by short incubations with propidium iodide (PI) due to extensive loss of cell membrane integrity, whereas cells undergoing apoptosis show a much lower uptake of PI than necrotic cells. By using PI staining together with Annexin V staining it is therefore possible to distinguish living (i.e., Annexin V^−^ PI^−^), early apoptotic (i.e., Annexin V^+^ PI^−^), late apoptotic (i.e., Annexin V^+^ PI^dim^) and late apoptotic/necrotic (i.e., Annexin V^+^ PI^bright^) cell populations from each other (Overbeeke et al., 1998; Vitale et al., 1993). When taking a closer look at the Annexin V^+^ PI^+^ Jurkat T-cells in the upper right quarter, an Annexin V^+^ PI^dim^ (grey arrow) and an Annexin V^+^ PI^bright^ (black arrow) cell population could be detected, indicating the occurrence of a late apoptotic and a late apoptotic/necrotic cell population, respectively (Fig. 4D).

In contrast to the crude extract derived from *Pseudomonas* sp. In5, treatment of the Jurkat T-cells with 2.5 µg solid/ml of the *Pseudomonas* sp. Gr1 derived crude extract showed a much weaker induction of Annexin V staining with a mean value of 15.2% Annexin V positive cells  compared to the 95.0% induction by the *Pseudomonas* sp. In5 derived crude extract (Figs. 4C and 4D). These results suggest that the anticancer effect of the *Pseudomonas* sp. In5 derived crude extract involves the activity of nunamycin.

### Nunamycin and nunapeptin purified from the Pseudomonas sp. In5 derived crude extract display distinct antimicrobial activities and a synergistic anticancer activity

To test the antimicrobial and anticancer activities of the two NRPs, nunamycin (*m*/*z* 1,138) and nunapeptin (*m*/*z* 2,023–2,075), we purified the NRPs from the *Pseudomonas* sp. In5 derived crude extract by HPLC. We observed distinct antimicrobial activities against *R. solani* and *P. aphanidermatum* between nunamycin and nunapeptin, which were comparable to our previous observations (Fig. 2A and Michelsen et al., 2015). Nunamycin potently inhibited mycelial radial growth of *R. solani* with a mean value of 52% inhibition, whereas no growth inhibition of *P. aphanidermatum* was observed (Fig. 2A). In contrast, nunapeptin strongly inhibited the growth of *P. aphanidermatum* with a mean value of 48% inhibition, but did not affect the growth of *R. solani* (Fig. 2A).

**Figure 6:**
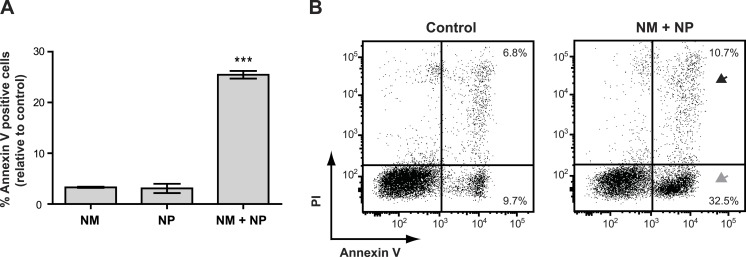
Annexin V staining of cancer cells after treatment with purified nonribosomal peptides. Annexin V staining without (A) or with (B) PI staining of the Jurkat T-cell cancer cell line was examined by flow cytometry after treatment with DMSO (control), or 10 µg/ml nunamycin (NM) and nunapeptin (NP) alone or in combination (NM + NP). (A) The bar graph show the % of apoptotic cells above the control level (mean ± SEM; *n* = 4). ^∗∗∗^*p* < 0.001 (students *t*-test). (B) The grey arrowhead shows early apoptotic cells (Annexin V^+^, PI^−^), whereas the black arrowhead shows late apoptotic/necrotic cells (Annexin V^+^, PI^+^). The *Y*-axis shows PI staining (in fluorescence units) and the *X*-axis shows Annexin V staining (in fluorescence units), both in log scale. Data is representative of four independent experiments.

Surprisingly, we found that treatment with nunamycin alone was not able to induce apoptosis in the Jurkat T-cell line (Fig. 6A), despite the strong indications from our investigations with the crude extracts. In addition, no anticancer activity was observed by treatment with nunapeptin alone (Fig. 6A). However, when the two NRPs were combined, a significant induction of Annexin V staining in the cancer cells was observed with a mean value of 25.5% Annexin V positive cells relative to the control treatment (Fig. 6A), which included an increase in both early (i.e., 22.8% Annexin V^+^, PI^−^) and late (i.e., 3.9% Annexin V^+^, PI^dim/bright^) apoptotic cells (Fig. 6B). Thus, despite the individual potent antimicrobial activities observed with nunamycin and nunapeptin, treatment with the two compounds simultaneously was essential to obtain robust anticancer activity. Accordingly, a synergistic anticancer effect between nunamycin and nunapeptin would explain the much lower induction of Annexin V staining of the Jurkat T-cells observed by the crude extract derived from the nunamycin deficient strain *Pseudomonas* sp. Gr1 (Figs. 4C and 4D).

## Discussion

Bioactive metabolites are produced by microorganisms in the soil as a defense mechanism against soil borne pathogenic microorganisms invading the microenvironment (Raaijmakers & Mazzola, 2012; Weller, 1988). However, as some of these naturally derived bioactive metabolites are directed against eukaryotic fungal and oomycete pathogens, they can also eradicate various infectious diseases in humans and function as chemotherapy agents as well as immunosuppressant drugs (Cameotra & Makkar, 2004; Demain & Sanchez, 2009). Species of *Pseudomonas* are common bacterial inhabitants of the soil environment, and they produce a wide variety of metabolites with bioactivities, including enzymes, volatiles, toxins and NRPs (Haas & Defago, 2005; Weller, 2007). We have previously isolated the bacterium, *Pseudomonas* sp. In5, from a Greenlandic potato soil, capable of producing various bioactive metabolites, including hydrogen cyanide and the two NRPs, nunamycin and nunapeptin (Michelsen & Stougaard, 2011; Michelsen & Stougaard, 2012; Michelsen et al., 2015). So far, our investigations have primarily focused on identifying the antimicrobial potentials of *Pseudomonas* sp. In5, a bioactivity that was also verified in the present study. In this study we further expand our knowledge of the bioactivities by *Pseudomonas* sp. In5, providing new evidence that a crude extract derived from *Pseudomonas* sp. In5 possesses anticancer potential. We found that the crude extract derived from *Pseudomonas* sp. In5 was able to increase staining of three different cancer cell lines (i.e., T-cell leukemia, mantle cell leukemia, and melanoma) with the apoptotic marker Annexin V. In agreement with our previous studies (Michelsen et al., 2015), we observed that nunamycin and nunapeptin is important for the antimicrobial activity observed by *Pseudomonas* sp. In5. However, even though nunamycin or nunapeptin treatment alone show robust, albeit distinct, antimicrobial activities, the anticancer effect seems to involve a synergistic effect between the two compounds, as Annexin V staining was induced in the cancer cells only after treatment with the two compounds in combination.

Nunamycin and nunapeptin show structural similarities to the NRPs syringomycin and syringopeptin produced by *P. syringae* pv. *syringae* B728a (Michelsen et al., 2015). The proposed mode-of-action of syringomycin and syringopeptin has been ascribed to their pore-forming ability in the lipid bilayer of cell membranes resulting in massive ion fluxes that are detrimental to cells (Bender, Alarcón-Chaidez & Gross, 1999). Fungal hyphae exposed to such cellular stresses, initially accumulate vacuoles and show abnormal growth before their growth halts completely (Raaijmakers et al., 2010; Van Peer et al., 2009). In the present study, we focus on identifying new bioactivities of *Pseudomonas* sp. In5 and therefore do not provide any detailed mechanistic effects of the crude extract. However, based on the nature of nunamycin and nunapeptin residing in the *Pseudomonas* sp. In5 crude extract, we hypothesize that the high vacuolization and abnormal formation of mitochondria observed in the *R. solani* Ag3 hyphae after exposure to the crude extract could be due to membrane damage from the activity of nunamycin and nunapeptin ultimately resulting in a mycelial growth inhibition.

To study the anticancer activity of the *Pseudomonas* sp. In5 derived crude extract, we stained the cells with the apoptotic marker Annexin V with or without PI. This staining method is a reliable tool to detect early and late apoptotic cells by flow cytometry (Overbeeke et al., 1998; Silva, 2010; Vermes et al., 1995; Vitale et al., 1993). In this study we found that the magnitude of anticancer activity against Jurkat T-cells differed between the *Pseudomonas* sp. In5 crude extract and the purified nunamycin and nunapeptin in combination. After treatment with the *Pseudomonas* sp. In5 crude extract the majority of Jurkat T-cells seemed to be in a late apoptotic (i.e., Annexin V^+^ PI^dim^) or late apoptotic/necrotic (i.e., Annexin V^+^ PI^bright^) state, whereas after treatment with nunamycin and nunapeptin we detected an early apoptotic cell population (i.e., Annexin V^+^ PI^−^) and only very few late apoptotic cells/necrotic cells (i.e., Annexin V^+^ PI^dim/bright^). The observed difference in magnitude could indicate that additional bioactive metabolites are present in the *Pseudomonas* sp. In5 crude extract positively affecting the anticancer activity.

Furthermore, the content of phospholipids, proteins and sterols in a cell membrane differs depending on the organism, cell type, and the nature of the membrane and thus can affect the bioactivities of microbial derived peptides (Hoskin & Ramamoorthy, 2008). Therefore, fundamental differences between the cell membranes of cancer cells and normal cells (Hoskin & Ramamoorthy, 2008) could account for the ability of the *Pseudomonas* sp. In5 derived crude extract to affect the viability of the cancer cells tested while sparing naïve and activated CD4 T-cells.

Many current chemotherapeutic regimens induce systemic cell death, leaving a weakened immune system unable to adequately target cancer antigens (Kroemer et al., 2013). However, it has become widely accepted that an effective immune response is needed to completely eradicate cancers following treatment with chemotherapeutic agents that trigger cell death (Lake & Robinson, 2005). Therefore, the cell specificity and the retention of T cells observed with the *Pseudomonas* sp. In5 derived crude extract in this study is intriguing, although the examination of immunogenic cell death is beyond the scope of this work.

Investigations of crude extracts by a biobased approach with arbitrary testing of isolated metabolites has traditionally been used to detect new potential drug candidates (Singh & Pelaez, 2008). However, the isolation and identification of bioactive components from complex extracts can be rather time-consuming (Singh & Pelaez, 2008). In this study, we demonstrate that a multi-component crude extract may possess bioactivities as a result of synergy between two or more bioactive metabolites challenging the workflow of the biobased approach even further. Using molecular techniques to construct bioactive deficient mutants combined with biobased screening of the derived crude extracts thus might be a more efficient approach to lead the discovery of new single-acting or synergistic drug candidates.

## Supplemental Information

10.7717/peerj.1476/supp-1 Supplemental Information 1 Antimicrobial activities of *Pseudomonas* crude extracts and purified nonribosomal peptides (A) The antimicrobial effect of the crude extracts derived from wild type *Pseudomonas* sp. In5 (In5 ex) or the nunamycin knock-out mutant, Gr1 (Gr1 ex) as well as the purified NRPs, nunamycin (NM) or nunapeptin (NP) was examined against the basidiomycete, *R. solani* AG3 (light grey bars), or oomycete, *P. aphanidermatum* (dark grey bars). Antimicrobial activity was scored as percent inhibition of radial growth (PIRG) (mean ± SEM, *n* = 3).

10.7717/peerj.1476/supp-2 Supplemental Information 2 Induction of apoptosis in cancer cell lines after treatment with *Pseudomonas* crude extracts Induction of apoptosis in the (A) melanoma, (B) Granta and (C) Jurkat T-cells by crude *Pseudomonas* extracts and FR901228 (FR).

## Additional Information and Declarations

### Data Availability

The following information was supplied regarding data availability: Raw data sets for Fig. 2A as well as Figs. 4 and 6A have been uploaded as Supplemental Information 1 and Supplemental Information 2, respectively.
